# Association of PAEs with Precocious Puberty in Children: A Systematic Review and Meta-Analysis

**DOI:** 10.3390/ijerph121214974

**Published:** 2015-12-01

**Authors:** Yi Wen, Shu-Dan Liu, Xun Lei, Yu-Shuang Ling, Yan Luo, Qin Liu

**Affiliations:** School of Public Health and Management, Center for Medicine and Social Development, Innovation Center for Social Risk Governance in Health, Chongqing Medical University, Chongqing 400016, China; wen77820043@163.com (Y.W.); liushudankelly@163.com (S.-D.L.); leixun521@163.com (X.L.); lvylpwy@163.com (Y.-S.L.); ly987146114@163.com (Y.L.)

**Keywords:** phathalic acid esters, precocious puberty, systematic review, meta-analysis

## Abstract

*Background*: Precocious puberty (PP) currently affects 1 in 5000 children and is 10 times more common in girls. Existing studies have tried to detect an association between phathalic acid esters (PAEs) and PP, but the results did not reach a consensus. *Objective*: To estimate the association between PAEs and children with PP based on current evidence. *Methods*: Databases including PubMed (1978 to March 2015), OVID (1946 to March 2015), Web of Science (1970 to March 2015), EBSCO (1976 to March 2015), CNKI (1979 to March 2015), WANFANG DATA (1987 to March 2015), CBM (1978 to March 2015) and CQVIP (1989 to March 2015) were searched to identify all case-control studies that determined the exposure and concentration of PAEs and their metabolites in children with PP. Meta-analysis of the pooled standard mean difference (SMD) and odds ratio (OR) with 95% confidence intervals (CI) were calculated. *Results*: A total of 14 studies involving 2223 subjects were finally included. The pooled estimates showed that PP was associated with di-(2-ethylhexyl)-phthalate (DEHP) exposure (OR: 3.90, 95% CI: 2.77 to 5.49). Besides, the concentration of DEHP (SMD: 1.73, 95% CI: 0.54 to 2.91) and di-*n*-butyl phthalate (DBP) (SMD: 4.31, 95% CI: 2.67 to 5.95) in the PP group were significantly higher than those in the control group, respectively, while no difference was detected between case and control groups in either serum or urinary concentration of mono-(2-ethylhexyl)-phthalate (MEHP), monobutyl phthalate (MBP), mono(2-ethyl-5-oxohexyl) phthalate(MEOHP), mono-(2-ethyl-5-carboxypentyl) phthalate (MECPP), monomethyl phthalate (MMP), monobenzyl phthalate (MBzP) or monoethyl phthalate (MEP). *Conclusions*: Exposure of DEHP and DBP might be associated with PP risk for girls, however, there is no evidence to show an association between the exposure to most PAE metabolites and PP. Given the moderate strength of the results, well-designed cohort studies with large sample size should be performed in future.

## 1. Introduction

Precocious puberty (PP) currently affects 1 in 5000 children and is 10 times more common in girls [1]. Previous studies [2] have reported that the overall PP incidence continues to increase and the puberty development age has shown an advancing trend since the 19th century. Advanced progression of secondary sex characteristics occurs in children with PP leading to compromised adult height, poor social adaptability and emotional disorders. Meanwhile, the risk of endocrine disease and even cancer can sharply increase for a child if hormones stay at an abnormal level for a long time [2,3]. The cause of precocious puberty may be associated with certain conditions such as infections, tumors, brain abnormalities, histiocytosis, radiation, injuries, or the exposure to environmental endocrine disruptors [2]. The phthalic acid esters (PAEs) are one of the classes of environmental endocrine disruptors (EEDs) involved in many endocrine diseases, and studies have argued that exposure to PAEs may be associated with PP [4]. Population studies have indicated that most children are exposed to PAEs. A German study collected the morning urine of 254 children aged 3–14 years from March 2001 to March 2002, and the average concentrations of the primary metabolite MEHP, and secondary metabolites 5 OH-MEHP, and 5 oxo-MEHP were 7.9, 52.1 and 39.9 μg/L, respectively, indicating that children were not free of PAE exposure [5]. The U.S. NHANES study from 1999 to 2000 found measurable concentrations of MEP, MBP, and MBzP in more than 97% of the 2540 urine samples tested, indication that exposure to PAEs is widespread in the United States [6]. Animal studies have confirmed that the mechanism of action of PAEs and their metabolites is to act as anti-androgens, but DBP also has comparatively strong estrogenic properties [7] which may lead to reproductive disruption [8] in females including reproductive maturation [9,10]. However, existing human studies don not seem to have achieved a consensus so far. Colon *et al*. [11] observed significantly higher levels of PAEs in 31 Puerto Rican girls with early thelarche compared to controls, which suggested a possible association between PAEs and premature thelarche, whereas, a study on U.S. girls reported phthalate levels in urine were not related with central precocious puberty of pre-pubescent girls [12]. Therefore, it is necessary to perform a systematic review and meta-analysis to synthesize current study evidence and identifying the association between PAEs and children with PP.

## 2. Methods

### 2.1. Selection Criteria

Inclusion criteria: only cohort studies and case-control studies that determine the exposure and concentrations of PAEs and their metabolites in children with PP including central precocious puberty (CPP), peripheral precocious puberty (PPP) and premature thelarche (PT) were identified. In our study, PP is defined as the appearance of secondary sex characteristics before the age of eight years in girls and nine years in boys, increased growth velocity, advanced bone maturation, pubertal LH response in a standard GnRH test (100 ug iv) [3,13]_._

Exclusion criteria: (1) any other disease that would affect endocrine levels such as McCune Albright syndrome, adrenal cortical tumors, hypothalamic hamartoma; (2) certain types of publication such as news items, letters, editorials, comments, reviews, conference summary or animal studies.

### 2.2. Search Strategy

We searched PubMed (1978 to March 2015), OVID (1946 to March 2015), Web of Science (1970 to March 2015), EBSCO (1976 to March 2015), CNKI (1979 to March 2015), WANFANG DATA (1987 to March 2015), CBM (1978 to March 2015) and CQVIP (1989 to March 2015) using both the MeSH terms and free terms “endocrine disruptors” or “EEDs” or “endocrine disruptors chemicals” or “EDCs” or “PAEs” or “DEHP” or “DBP” “DEP” or “DEP” or “DMP” or “MBP” or “MEHP”, in combination with “sexual precocity” or “precocious puberty” or “spermatorrhea” or “menarche”. Appropriate modification was adapted up to requirements over different databases (detailed search terms see Supplementary Table S1). All the retrieved publications were entered into reference-managing software (EndNote, version X6, Thomson Scientific, Stamford, CT, USA).

### 2.3. Data Screening and Extraction

Two authors (Yi Wen, Yu-Shuang Ling) screened all the retrieved literature by title, abstract and full texts using criteria mentioned above. Cross-checking was conducted for accuracy. Disagreements were resolved through discussion or consultation with the third author (Qin Liu) to a consensus. The following data were extracted from included studies using a pre-designed extraction form: (1) general information, including authors, publication year, country; (2) study design; (3) participants characteristics and sample size; (4) outcomes, consist of the exposure and concentrations of different types of PAEs and their metabolites; (5) detection methods and instrument.

### 2.4. Risk of Bias Assessment

The Newcastle-Ottawa Scale (NOS) [14] was used to assess the methodology quality of included case-control studies. Assessment items included the selection of cases (four items, four scores), comparability of cases and controls (one item, two scores), and ascertainment of exposure to risks (three items, three scores). Research scored 0~3 was considered as low quality, scored 4~6 as moderate quality, while scored 7~9 as high quality, respectively [15]. The quality scores did not affect studies inclusion, but would be considered when performing sensitivity analysis and interpreting our research results.

### 2.5. Statistical Analysis

Meta-analysis was performed using Review Manager Software (Version5.2, Cochrane Collaboration, London, UK) and Stata Software (Version12.0, StataCorp, College Station, TX, USA). The outcomes of continuous and dichotomous variables were assessed as standard mean difference (SMD) and odds ratio (OR) with 95% confidence intervals (CI), respectively. Results reported in median and range was converted to mean and standard deviation [16]. Heterogeneity among the results of the included studies was evaluated by χ^2^ and *I*^2^ statistic tests. Results were considered to be statistically significant when *p* < 0.05. Once effects were found to be heterogeneous (*I*^2^ > 50% or *p* < 0.05), the random effects model was used. Otherwise, the fixed effects model was used. Considering the different exposure levels of PAEs in different countries, subgroup analysis on different countries was conducted in conditions of plenty included studies. All the outcome analyses were sensitivity assessed using the leave-one-out approach for the stability of the result. Since the number of included trials was less than 10 in every outcome, we did not assess the publication bias [17].

## 3. Results

### 3.1. Search Results

**Figure 1:**
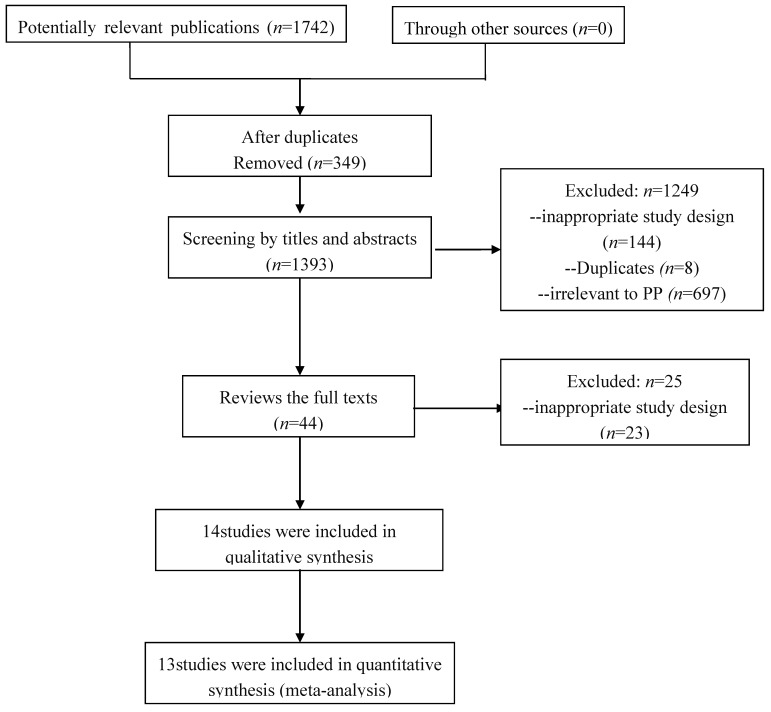
Flow Diagram of the Literature Search.

Among 1742 records identified from the eight databases, a total of 14 studies [18,19,20,21,22,23,24,25,26,27,28,29] involving 1111 PP girls and 1112 normal control girls were finally identified through a strict screening process (Figure 1).

### 3.2. Characteristics of Included Studies

Table 1 presents the general characteristics of included studies. All the studies were published between 2000 and 2015, with the sample size ranging from 56 to 488. Among those 14 studies, ten studies were conducted in China, while the other four studies were conducted in Korea, USA, Denmark and Puerto Rico, respectively. Blood test was conducted in ten studies and urinary test was conducted in the other four studies. All studies were case-control designs, and the controls were mainly healthy populations which were matched with the PP group in age, gender, ethnicity and living environments. All subjects in all included studies were girls.

Among the ten studies conducted with blood tests, seven studies exclusively measured the exposure and concentration of three types of parent PAEs including DEHP, DBP, diethyl phthalate (DEP), two studies measured the concentration of both parent PAEs and metabolites including DEHP, DBP, DiBP, MEHP and MBP, only one study exclusively measured the concentration of metabolites including mono(2-ethyl-5-hydroxyhexyl) phthalate (MEHHP), MEOHP, mono-3-carboxypropyl phthalate (MCPP), MECPP, monoisobutyl phthalate (MiBP). For the other four studies conducted with urinary test, three studies exclusively measured eight kinds of PAE metabolites including MEHP, MBP, MMP, MBzP, MEP, monobutyl phthalate (MBuP), MEHHP and MEOHP, only one study measured the concentration of both parent PAEs and metabolites including DEHP, MEP, MBP, MBzP. Categories of PAEs and metabolites in each study with blood or urine test are shown in Table 1.

### 3.3. Risk of Bias in Included Studies

Among 14 included studies, except for seven studies were considered as moderate risk of bias (scores of 5 and 6), the others were assessed as low risk of bias (scores of 7 and 8) (Table 2). All studies had adequate case and controls definition, and exposure assessment. No standard method was adopted in sampling, which would affect the representativeness of the cases. The controls were recruited from the same community as cases in only five studies. Seven just matched case and control by age without consideration of other confounding factors, while one article did not mention measure to control for confounding factors.

**Table 1:** Characteristics of studies included in the meta-analysis.

Study	Country	Test Specimen	Sample SizePP/Control	Kinds of PP	Age		Categories of PAEs and Metabolites	Detection Methods	Instrument
					PP	Control			
Chou *et al.* [18]	China	urine	30/33	PT	6.7±1.8	8.2±1.3	MEHP, MMP, MBzP, MBuP	HPLC-ESI-MS/MS	NA
Yum *et al.* [19]	Korea	blood	150/90	PP	8.91±1.40	8.5±1.68	DEHP,DBP,MEHP,MBP	HPLC	NA
Cai *et al.* [20]	China	blood	110/100	ICPP	6.1~11.3	6.5~11.2	DEHP,DBP	GC	AL 3000
Ke *et al.* [21]	China	blood	123/102	ICPP	5.6~11.3	6.2~11.1	DEHP,DBP	HPLC	NA
Lin [22]	China	urine	32/20	ICPP	6.92 (1.8~8.5)	5.92 (2.17~8.17)	MEHP, MBP, MMP, MBzP, MEP	HPLC-MS/MS	HP-1100
Lu *et al.* [23]	China	blood	73/38	PP	5.8~11.9	6.5~11.2	DEHP	HPLC	HP-1100
Wang *et al.* [24]	China	blood	50/50	PT	1.04±0.53	1.12±0.65	DEP	HPLC	Waters 1525
Chen *et al.* [25]	China	urine	73/31	ICPP	2.5~11.5	2.2~8.3	MEHP, MBP, MMP, MBzP, MEP,MEHHP,MEOHP	HPLC	HPLC 1200
Lomenick *et al.* [12]	USA	blood	28/28	ICPP	7.24±0.24	7.12±0.25	MEHP, MBP, MBzP, MEP, MEHHP, MEOHP,MCPP, MECPP, MiBP	HPLC	NA
Colon *et al.* [11]	Puerto Rico	blood	41/35	PT	0.5~8	0.5~10	DBP	GC/MS	NA
Frederiksen *et al.* [26]	Denmark	urine	24/184	CPP or PT	7.4~9.9	7.4~9.9	DEHP, MEP, MBP, MBzP	LCMS/ MS	NA
Zhang *et al.* [27]	China	blood	80/50	ICPP	6~11	6~12	DEHP	HPLC	HP-1100
Song *et al.* [28]	China	blood	80/80	PP	5~7	5~7	DEHP,DBP	GC	GC5890F
Yang *et al.* [29]	China	blood	217/271	ICPP	7.91±0.85	7.20±0.70	DEHP, DBP, MBP, DiBP	HPLC-MS/MS	NA

PT-premature thelarche; CPP-central precocious puberty; ICPP-idiopathic central precocious puberty; EP-early normal puberty. AL3000 (Agilent, California, CA, USA); HP-1100, Waters 1525, HPLC 1200 (Waters, Massachusetts, MA, USA); GC5890F (Shimadzu, Kyoto, Japan).

**Table 2:** The Newcastle-Ottawa Scale (NOS) for assessing the methodology quality.

Study	Selection				Comparability	Exposure			Score ^a^
	Adequate Case Definition	Representativeness of the Cases	Selection of Controls	Definition of Controls	Comparability of Cases and Controls on the Basis of the Design or Analysis	Ascertainment of Exposure	Same Method of Ascertainment for Cases and Controls	Non-Response Rate	
Chou *et al.* [18]	1	0	1	1	1	1	1	1	7
Yum *et al.* [19]	1	0	0	1	1	1	1	1	6
Cai *et al.* [20]	1	0	0	1	1	1	1	1	6
Ke *et al.* [21]	1	0	0	1	1	1	1	1	6
Lin [22]	1	0	1	1	2	1	1	1	8
Lu *et al.* [23]	1	0	0	1	0	1	1	1	5
Wang *et al.* [24]	1	0	0	1	2	1	1	1	7
Chen *et al.* [25]	1	0	1	1	2	1	1	1	8
Lomenick *et al.* [12]	1	0	0	1	2	1	1	1	7
Colon *et al.* [11]	1	0	0	1	1	1	1	1	6
Frederiksen *et al.* [26]	1	0	1	1	2	1	1	1	8
Zhang *et al.* [27]	1	0	0	1	1	1	1	1	6
Song *et al.* [28]	1	0	1	1	2	1	1	1	8
Yang *et al.* [29]	1	0	0	1	1	1	1	1	6

a: Low-quality research, scored 0–3; moderate quality research, scored 4–6; high-quality research, scored 7–9.

### 3.4. Serum PAEs and PP Risk

#### 3.4.1. DEHP Exposure and PP Risk

**Figure 2:**
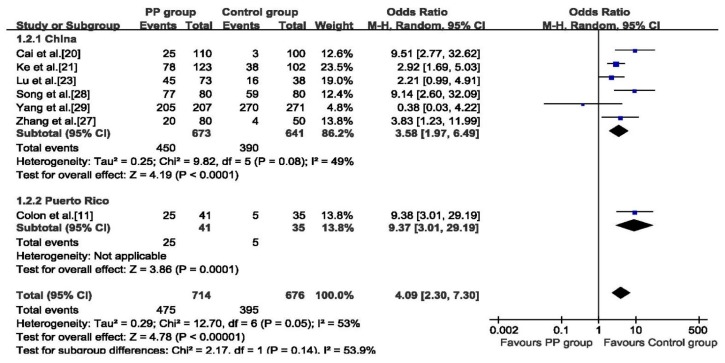
Forest plot for the association between serum DEHP and PP.

Meta-analysis based on 1390 subjects indicated that DEHP exposure was a risk factor of PP with a pooled OR of 4.09 (95% CI: 2.30 to 7.30). Random effects model was adopted in addressing the association between DEHP and PP (*p* = 0.05, *I*^2^ = 53%), (Figure 2). The subgroup analysis was inconsistent with the overall pooled estimate, studies conducted in China showed that DEHP was a risk factor of PP (OR: 3.58, 95% CI: 1.97 to 6.49) and studies from Puerto Rico showed an even higher OR of 9.37 (95% CI: 3.01 to 29.19). In addition, the association between DEHP and PP was not changed in each individual sensitivity analysis by leaving one out approach (see Supplementary Material Figure S1).

#### 3.4.2. DBP Exposure and PP Risk

**Figure 3:**
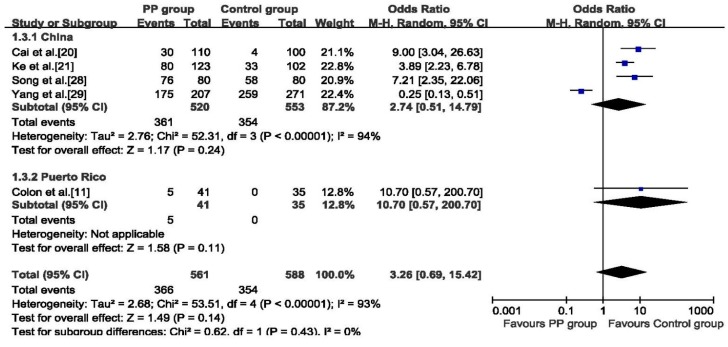
Forest plot for the association between serum DBP and PP.

No evident association was found between DBP exposure and PP with a pooled OR of 3.26 (95% CI: 0.69 to 15.42) for 1149 girls, the random effects analysis was adopted because of heterogeneity among studies (*p*< 0.01, *I*^2^ = 93%), (Figure 3). The subgroup analysis on China’s studies also showed DBP might not be a risk factor for PP (OR: 2.74, 95% CI: 0.51 to 14.79). After removing Yang 2014 [29] in sensitivity analysis, the pooled result substantially altered (OR: 5.07, 95% CI: 3.24 to 7.93) (see Supplementary Material Figure S2).

#### 3.4.3. DEP

Only one study [24] reported the exposure of DEP with 50 girls in both PP and control group. The results of the study showed that DEP exposure was associated with PP (OR: 93.38, 95% CI: 5.46 to 1596.86).

### 3.5. Serum Concentration Difference of PAEs and Their Metabolites between PP Group and Control Group

#### 3.5.1. DEHP

**Figure 4:**
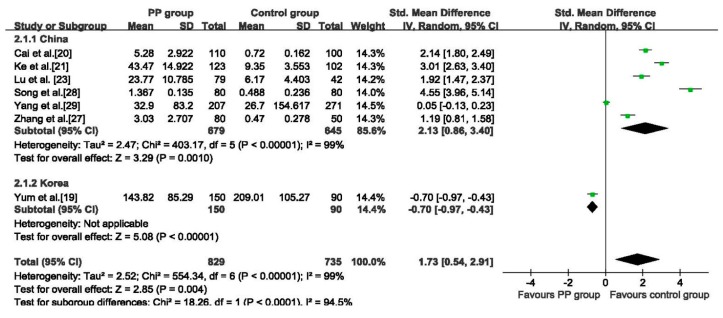
Forest plot for serum concentration of DEHP between PP group and control group.

Seven studies [19,20,21,23,27,28,29] reported the serum concentration of DEHP, showing that the concentration of DEHP detected in the PP group was higher than that in control group (SMD: 1.73, 95% CI: 0.54 to 2.91, 1564 girls), the random effects model was adopted because of the high heterogeneity (*p* < 0.01, I^2^ = 99%). In subgroup analysis based on different countries, the PP group had higher DEHP concentration than the control group in six Chinese studies (SMD: 2.13, 95% CI: 0.86 to 3.40, 1324 girls), while the Korean study showed a reverse result (SMD: −0.70, 95% CI: −0.97 to −0.43, 240 girls) (Figure 4). The higher concentration of DEHP detected in PP group was not changed in sensitivity analysis (see Supplementary Material Figure S3).

#### 3.5.2. DBP

**Figure 5:**
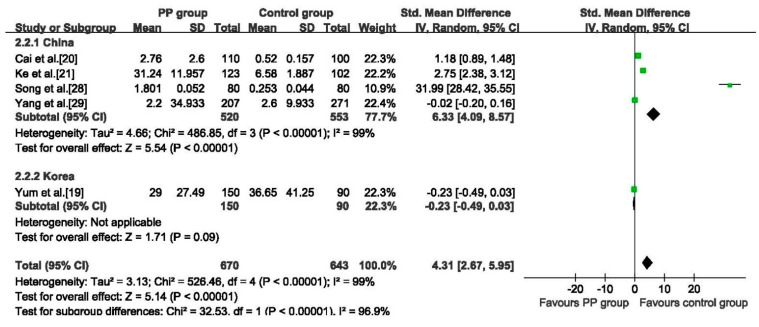
Forest plot for serum concentration of DBP between PP group and control group.

Five studies [19,20,21,28,29] reported the serum concentration of DBP. The pooled data showed that the concentration of DBP detected in the PP group was higher than that in the control group (SMD: 4.31, 95% CI: 2.67 to 5.95, 1313 girls), the random effects analysis was adopted because of heterogeneity among studies (*p* < 0.01, *I*^2^ = 99%). In subgroup analysis, pooled data of four Chinese studies revealed that the PP group had higher DEHP concentration than the control group (SMD: 6.33, 95% CI: 4.09 to 8.57, 1073 girls), while the Korean study showed no statistically differences between two groups (SMD: −0.23, 95% CI: −0.49 to 0.03, 240 girls), (Figure 5). When the result of Song 2014 [28] was removed in the sensitivity analysis, the pooled data changed to no statistically differences between the two groups. (Supplementary Material Figure S4).

#### 3.5.3. DiBP

Only one study [29] reported the serum concentration of DiBP. The results of the study demonstrated no significant difference between the two groups (SMD: –0.05, 95% CI: –0.23 to 0.13, 478 girls).

#### 3.5.4. MEHP

**Figure 6:**
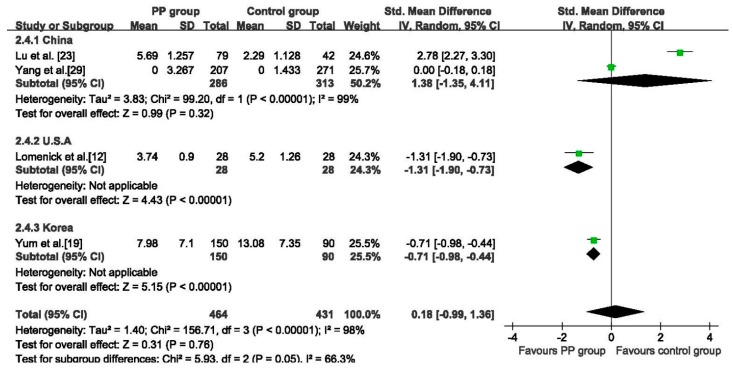
Forest plot for concentration of MEHP between PP group and Control group.

Data regarding serum concentration of MEHP were provided in four studies [12,19,23,29]. There was no significant difference between the two groups (SMD: 0.18, 95% CI: −0.99 to 1.36, 895 girls), the random effects analysis was adopted because of heterogeneity among studies (*p* < 0.01, *I*^2^ = 99%). In subgroup analysis, pooled data of the two Chinese studies showed no statistically significant differences between two groups (SMD: 1.38, 95% CI: −1.35 to 4.11, 599 girls), while in one America study (SMD: −1.31, 95% CI: −1.90 to −0.73) and one Korea study (SMD: −0.71, 95% CI: −0.98 to −0.44), the PP group had lower MEHP concentration than the control group (Figure 6). The concentrations of MEHP were not statistically different between the two groups in sensitivity analysis (see Supplementary Material Figure S5).

#### 3.5.5. MBP

**Figure 7:**
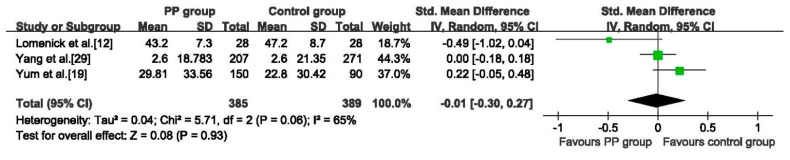
Forest plot for serum concentration of MBP between PP group and Control group.

Three studies [12,19,29] reported the serum concentration of MBP. The pooled data showed no significant difference between the two groups (SMD: −0.01, 95%CI: −0.30 to 0.27, 774 girls), the random effects model was adopted because of heterogeneity among studies (*p* = 0.06, *I*^2^ = 65%), (Figure 7). No difference between the two groups was detected in sensitivity analysis of the MBP concentration (see Supplementary Material Figure S6).

#### 3.5.6. Other PAE Metabolites

One study by Lomenick [12] reported five kinds of PAE metabolites in serum alone. The result showed that the concentration of MEHHP (SMD: −7.00, 95% CI: −11.82 to −2.18, 56 girls) and MiBP (SMD: −7.20, 95% CI: −10.21 to −4.19, 56 girls) detected in the PP group were lower than those in control group, while the concentration of MCPP detected in the PP group was higher than that in the control group (SMD: 1.21, 95% CI: 0.66 to 1.76, 56 girls) and no significant differences between the two groups with MEOHP (SMD: −2.40, 95% CI: −5.45 to 0.65, 56 girls) and MECPP (SMD: −4.80, 95% CI: −12.18 to 2.58, 56 girls).

#### 3.6. Urinary Concentration Difference of PAEs’ Metabolites between PP Group and Control Group

The pooled data showed no significant difference between the PP group and control group in urinary concentration of MEHP, MBP, MMP, MBzP and MEP (Table 3). Furthermore, the concentrations of MBP, MMP, MBzP and MEP between the two groups were still no different in sensitivity analysis (see Supplementary Material Figures S7–S11), while the pooled data showed that the PP group had a statistically lower MEHP concentration than the control group (SMD: −0.77, 95% CI: −1.40 to −0.14) when removing the result of Chen 2013 [25]. Only one study [18] reported the urinary concentration of MBuP, the results showed that the PP group had a statistically lower MBuP concentration than the control group (SMD: −0.77, 95% CI: −1.29 to −0.26, 63 girls). Another study [25] reported the urinary concentration of MEHHP and MEOHP, the results showed that the PP group had statistically higher concentration than the control group in both MEHHP (SMD: 0.76, 95% CI: 0.31 to 1.20, 100 girls) and MEOHP (SMD: 0.84, 95% CI: 0.39 to 1.28, 100 girls).

**Table 3:** Urinary concentration of five PAE metabolites between PP group and Control group.

Kinds of PAEs	Country	Combination Studies	Case/Contrl	SMD (95% CI)	*p*	Heterogeneity		
						χ^2^	*p*	*I*^2^
MEHP	China	3	129/82	−0.44 [−1.18, 0.29]	0.23	12.12	0.002	83%
MBP		3	127/233	−0.11 [−0.48, 0.26]	0.56	3.75	0.150	47%
	China	2	103/49	−0.21 [−0.87, 0.46]	0.54	3.46	0.060	71%
	Denmark	1	24/184	0.01 [−0.41, 0.44]	0.96			
MMP	China	3	129/82	0.27 [−0.21, 0.76]	0.28	5.51	0.060	64%
MBzP		4	153/266	0.00 [−0.43, 0.43]	0.99	9.46	0.020	68%
	China	3	129/82	−0.04 [−0.68, 0.59]	0.90	9.37	0.009	79%
	Denmark	1	24/184	0.09 [−0.33, 0.52]	0.68			
MEP		3	127/233	0.73 [−0.40, 1.86]	0.21	33.18	0.000	94%
	China	2	103/49	0.16 [−0.19, 0.50]	0.37	0.07	0.790	0%
	Denmark	1	24/84	1.84 [1.38, 2.30]	<0.01			

## 4. Discussion

EEDs are chemicals that either mimic or block hormones, thereby altering the normal hormone levels and endocrine function of the body [30]. EEDs such as dichlorodiphenyltrichloroethane (DDT) [31,32,33] and benzo(a)pyrene (BaP) [34,35]were proved to be associated with early puberty in both female rodents and humans. PAEs, as one of the EEDs often found in plastics and many cosmetic products, were confirmed to affect the female reproductive system and accelerate the onset of puberty in female rats [36,37]. However, the association between PAEs and children with PP has been somewhat inconsistent [11,12].

To improve limitations and adjust biases of individual studies, we performed a systematic review and meta-analysis to summarize association between PP and target PAEs and their metabolites, including DEHP, DBP, DEP, DiBP, MEHP, MBP, MEHHP, MEOHP, MCPP, MECPP, MiBP, MMP, MBzP, MEP and MBuP. The pooled estimates showed that serum DEHP exposure was associated with PP, and the serum concentrations of DEHP and DBP in PP group were significantly higher than those in the control group. According to the results of our meta-analysis, DEHP is a risk factor for PP and DBP might be related with PP given the instability results of sensitivity analysis. Only one study showed that DEP might be associated with PP. Therefore, we suggested that PAEs may have association with PP. One reason for the results may be that lots of PAEs were produced and used especially in developing countries every year [28], and DEHP, DBP as well as DEP account for the commonly used ones of plasticizer. That means, children in developing countries such as China and Puerto Rico have more opportunity to come into contact with DEHP, DBP and DEP. Moreover, since children’s metabolic pathways are immature, they are uniquely vulnerable to toxic chemicals in the environment [38]. Once exposed to PAEs, they may induce LH secretion by the pituitary, altering pubertal development and causing PP.

There is no significant difference in the concentration in both blood and urine of most PAE metabolites, including MEHP, MBP, MEOHP, MECPP, MMP, MBzP and MEP, between the children with PP and the controls. According to existing studies, although some PAEs have weak estrogenic activity *in vitro*, the corresponding PAEs metabolites have no estrogenic effects under the same testing conditions [39,40]. In addition, another study [41] also showed that some PAEs were bound to estrogen receptors *in vitro*, however, they had no uterotrophic effects and did not affect vaginal epithelial cell cornification in rats. Although there are no significant differences in MEHP, MBP, MEOHP, MECPP,MMP, MBzP and MEP between the children with PP and the controls in our included studies, the controls and the cases had various age, race, weight, height, and BMI, which could lead to the non-significant results in concentration of PAEs metabolites between the two groups. Anyway, we need more and higher quality evidence to prove that. Besides, the serum concentration of MEHHP and MiBP as well as urinary concentration of MBuP were lower in PP group than control group, while, the serum concentration of MCPP, urinary concentration of MEHHP and MEOHP were higher in PP group than control group. However, the differences need further study as all these results were reported in only one study with small sample size.

The lack of consistency between parent PAEs and metabolites associations with PP could be explained as follows: for one thing, there might be some potential for contamination associated with measurement of parent PAEs in serum, however we still tend to draw the conclusion that PAEs might be a potential risk for girls with PP from a statistical point of view. Previous studies showed that parent PAEs, such as DEHP and DBP, found in numerous medical devices, syringes and laboratory devices [42], may cause contamination during the process from blood sampling in the experiment procedure, leading to a relatively high exposure. Although seven out of nine included studies which measured the parent PAEs in serum used glass devices for all the experiments to avoid any potential contamination except for two studies which did not mention the laboratory device materials, there is still potential for laboratory contamination as well since phthalates are ubiquitous environmental contaminants. For another reason, it would not be possible to state that phthalate metabolites are not associated with PP due to the limited number of studies with small sample sizes included in the meta-analysis for each metabolite. In addition, phthalate metabolite measurements may outweigh the parent PAE measurements for the reasons that the contamination of serum sample of PAEs would be greatly minimized and they represent an integrative measure of exposure to phthalates from multiple sources and routes [43].

In the meta-analysis of serum DEHP exposure, the heterogeneity of the pooled estimate decreased after conducting subgroup analysis by country, which suggests that regional diversity is one of the potential sources of heterogeneity. Regarding the association of serum DEHP and DBP exposure and PP risk, consistent results were found in two subgroups, that is to say no regional differences were detected in the association between PP with DEHP or DBP. However, the pooled estimate of association between serum DBP exposure and PP changed from no difference (OR: 3.26, 95% CI: 0.69 to 15.42) to significant difference (OR: 8.31, 95% CI: 3.31 to 20.82) in the sensitivity analysis after removing Yang [29], which is attributed to it having the largest sample size with the most weight in this study and the fact its results were totally opposite to those of the other studies. One potential reason of the opposite result might be due to the interactions between chemicals. As the girls in Yang were exposed to 14 EEDs with weak estrogenic effects, the interaction of these different EEDs may enhance or weaken the estrogen effect [28]. As to serum concentration of DBP, the pooled data showed that the concentration of DBP detected in the PP group was higher than that in control group, and when the result of Song 2014 [28] was removed in the sensitivity analysis, the pooled data changed to no statistical difference. The likely reason for this change may be the highest standard mean difference between two groups the removed trial provided. For urinary concentration of MEHP, the pooled data showed that the PP group had statistically lower MEHP concentration than the control group when removing the result of Chen 2013 [25]. Except for Chen’s study, the other two studies showed that the control group had a significantly higher intake of seafood and use of plastic cups per month [18,22]. This may explain why the MEHP levels in the control group were higher than that in the PP groups.

Despite a rigorous systematic review and meta-analysis approach and relatively high quality of the studies included in the NOS assessment, some limitations should be considered when interpreting and expanding the present results. First, all the involved subjects of included studies were girls, which may affect the generality of research conclusions on PAEs exposure and PP risk for all children. One reason may be that the indicators of puberty in girls (breast bud development, pubic/axillary hair, menses onset) are more easily identified compared with boys (pubic/axillary, nocturnal emissions) and early stages of puberty in boys may be more subtle and require laboratory tests or standardized medical morphological tests. Second, heterogeneity that comes from regional diversity, race diversity, weight difference, sample size difference, age variation and health status, the concentration and duration of exposure of the study subjects or different detection methods, is relatively high for some meta-analysis and might be a potential effect when interpreting those synthesized results. After subgroup analysis conducted by region, the heterogeneity decreased to a large extent, indicating that different exposure levels of PAEs in different countries may be a more important factor in this case, and besides, it could also reflect the influence of race to some extent. Although most studies had shown that weight may be a factor influencing precocious puberty [44], we could not conduct the meta-analysis considering the girls’ weight as the included studies did not report the weights or BMI of the subjects. Third, the interpretations of association results are not always straightforward, because false-positive results (type *I* error) would be introduced when examining the association of phthalate metabolite with PP for children who exposed to multiple different phthalates. Meanwhile, type I error rate might be higher than 5% (using a *p*-value of 0.05 as the threshold for significance) because the sample sizes of most included studies were small (less than 100) [45]. Moreover, available existing evidence was so limited that the present review was restricted to only four most widely studied PAEs and eight metabolites among five countries based on 14 eligible studies, which might introduce potential bias to some extent.

## 5. Conclusions

To the best of our knowledge, the present work is the first attempt to determine the association between PAEs and PP by a systematic review and meta-analysis. From the combination results of individual studies, our findings suggested that a potential statistical association between phthalate exposure and PP, particularly, the exposure of DEHP and DBP might be a potential risk for girls with PP. No associations were identified between PP with MEHP, MBP, MEOHP, MECPP, MMP, MBzP or MEP. Nevertheless, it would be not appropriate to claim that those phthalate metabolites had no role in PP progress given the moderate strength of the present study. Well-designed epidemiological studies involving more types of PAEs with larger sample sizes are needed and measurement of phthalate metabolites is highly recommended for the examination of PAE exposure. Future studies should also employ methods to exclude potential contamination of samples during processing and analysis.

## Supplementary Files

Supplementary File 1
