# Supplementary material for: Association of PAEs with Precocious Puberty in Children: A Systematic Review and Meta-Analysis
Source: Int J Environ Res Public Health. 2015 Dec 1;12(12):15254–68. doi: 10.3390/ijerph121214974 (PMC4690910; doi:10.3390/ijerph121214974)
Supplement: Supplementary File 1 [file ijerph-12-14974-s001.pdf]

Supplementary Information

Association of PAEs with Precocious Puberty in Children: A Systematic Review and Meta-Analysis

Table S1. Search results.

Databases	Search Strategy	Records
CHKI	SU=(邻苯二甲酸+邻苯二甲酸酯+DEHP+DBP+DMP+DEP+MEHP+MBP+PAEs+环境内分泌干扰物+环境干扰物+EEDs+EDCs+邻苯二甲酸酯二甲酯+邻苯二甲酸酯二乙酯+邻苯二甲酸二丁酯+内分泌干扰物) AND SU=(性早熟+青春发动时相+月经初潮+首次遗精)	268
WanFang	题名或关键词:(邻苯二甲酸+邻苯二甲酸酯+DEHP+DBP+DMP+DEP+环境内分泌干扰物+环境干扰物+EEDs+EDCs+邻苯二甲酸酯二甲酯+邻苯二甲酸酯二乙酯+MEHP+MBP+PAEs+邻苯二甲酸二丁酯+环境内分泌物)*题名或关键词:(性早熟+青春发动时相+月经初潮+首次遗精)	127
VIP	(题名或关键词=邻苯二甲酸二丁酯 或 题名或关键词=环境内分泌物 与 范围=全部期刊) 或者 (题名或关键词=邻苯二甲酸酯二甲酯 或 题名或关键词=邻苯二甲酸酯二乙酯 或 题名或关键词=MEHP 或 题名或关键词=MBP 或 题名或关键词=PAEs 与 范围=全部期刊) 或者 (题名或关键词=DEP 或 题名或关键词=环境内分泌干扰物 或 题名或关键词=环境干扰物 或 题名或关键词=EEDs 或 题名或关键词=EDCs 与 范围=全部期刊) 或者 (题名或关键词=邻苯二甲酸 或 题名或关键词=邻苯二甲酸酯 或 题名或关键词=DEHP 或 题名或关键词=DBP 或 题名或关键词=DMP 与 范围=全部期刊) 与 (题名或关键词=性早熟 或 题名或关键词=青春发动时相 或 题名或关键词=月经初潮 或 题名或关键词=首次遗精 与 范围=全部期刊)	95
CBM	(((((((((((((((("邻苯二甲酸"[常用字段:智能]) OR "邻苯二甲酸酯"[常用字段:智能]) OR "DEHP"[常用字段:智能]) OR "DBP"[常用字段:智能]) OR "DMP"[常用字段:智能]) OR "DEP"[常用字段:智能]) OR "环境内分泌干扰物"[常用字段:智能]) OR "环境干扰物"[常用字段:智能]) OR "EEDs"[常用字段:智能]) OR "EDCs"[常用字段:智能]) OR "邻苯二甲酸酯二甲酯"[常用字段:智能]) OR "邻苯二甲酸酯二乙酯"[常用字段:智能]) OR "MEHP"[常用字段:智能]) OR "MBP"[常用字段:智能]) OR "PAEs"[常用字段:智能]) OR "邻苯二甲酸二丁酯"[常用字段:智能]) OR "环境内分泌物"[常用字段:智能] and ((("性早熟"[常用字段:智能]) OR "青春发动时相"[常用字段:智能]) OR "月经初潮"[常用字段:智能]) OR "首次遗精"[常用字段:智能]	74
Web of science	MeSH:((sexual precocity) OR MeSH:(precocious puberty) OR MeSH: (spermatorrhea) OR MeSH: (menarche) andMeSH: (endocrine disruptors chemicals) OR MeSH: (endocrine disruptors) OR MeSH: (EEDs) OR MeSH: (EDCs) OR MeSH: (DEHP) OR MeSH:(DBP) OR MeSH: (DMP) OR MeSH: (DEP) OR MeSH:(PAEs) OR MeSH: (MEHP) OR MeSH: (MBP))	501
OVID	((endocrine disruptors or EEDs or EDCs or DEHP or DBP or DEP or DMP or endocrine disruptors chemicals or PAEs or MBP or MEHP) and (sexual precocity or precocious puberty or spermatorrhea or menarche))	505
PubMed	(((((((((((endocrine disruptors) OR endocrine disruptor chemicals) OR eeds) OR edcs) OR dehp) OR DBP) OR DMP) OR DEP) OR PAEs) OR MEHP) OR MBP)) AND ((((sexual precocity) OR precocious puberty) OR spermatorrhea) OR menarche)	72
EBSCO	(endocrine disruptors or EEDs or EDCs or DEHP or DBP or DEP or DMP or endocrine disruptors chemicals or PAEs or MBP or MEHP) and (sexual precocity or precocious puberty or sermatorrhea or menarche)	90
Total		1742

DEHP: di-(2-ethylhexyl)-phthalate; DBP: di-n-butyl phthalate; DMP: di-methyl phthalate; DEP: diethyl phthalate; MEHP: mono-(2-ethylhexyl)-phthalate ; MBP: monobutyl phthalate ; PAEs: phathalic acid esters; EEDs: environmental endocrine disruptors; EDCs: endocrine disruptors chemicals. We have added a sentence (The results were searched by Chinese in CHKI, WanFang, VIP and CBM databases) under the Table S1 in the supplementary.

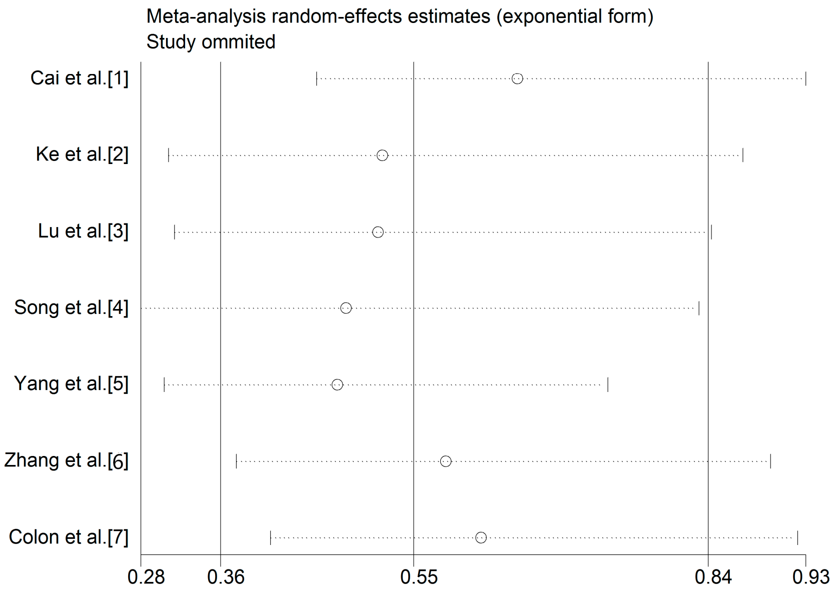

Figure S1. Serum DEHP exposure.

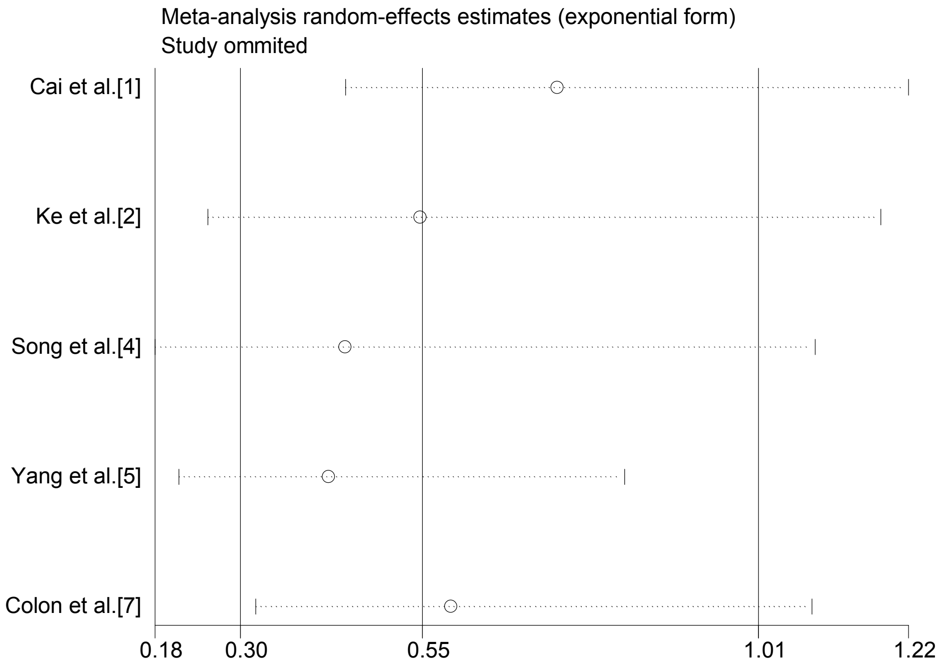

Figure S2. Serum DBP exposure.

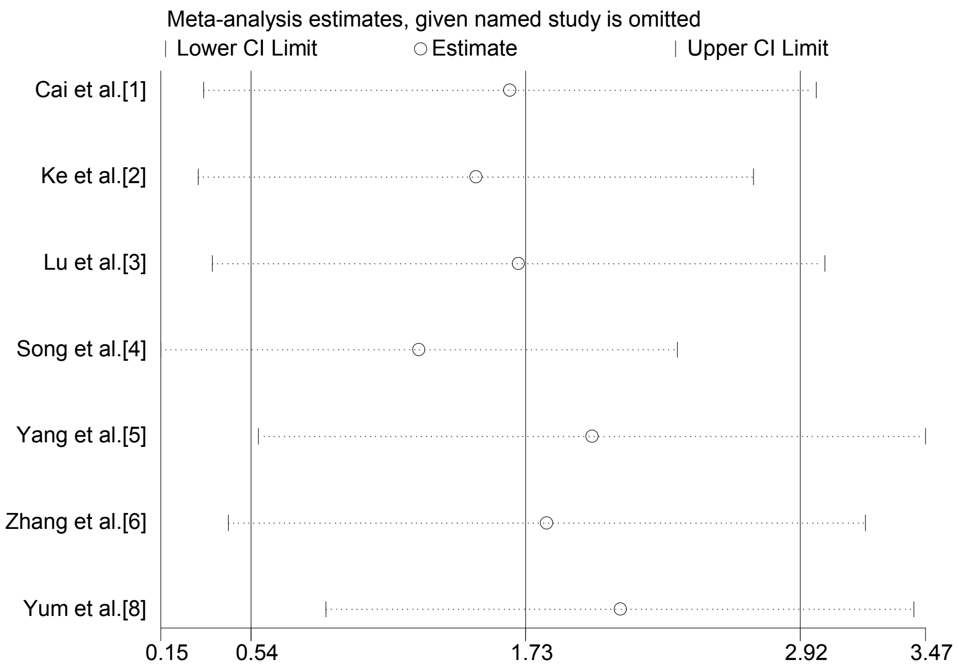

Figure S3. Serum DEHP concentration.

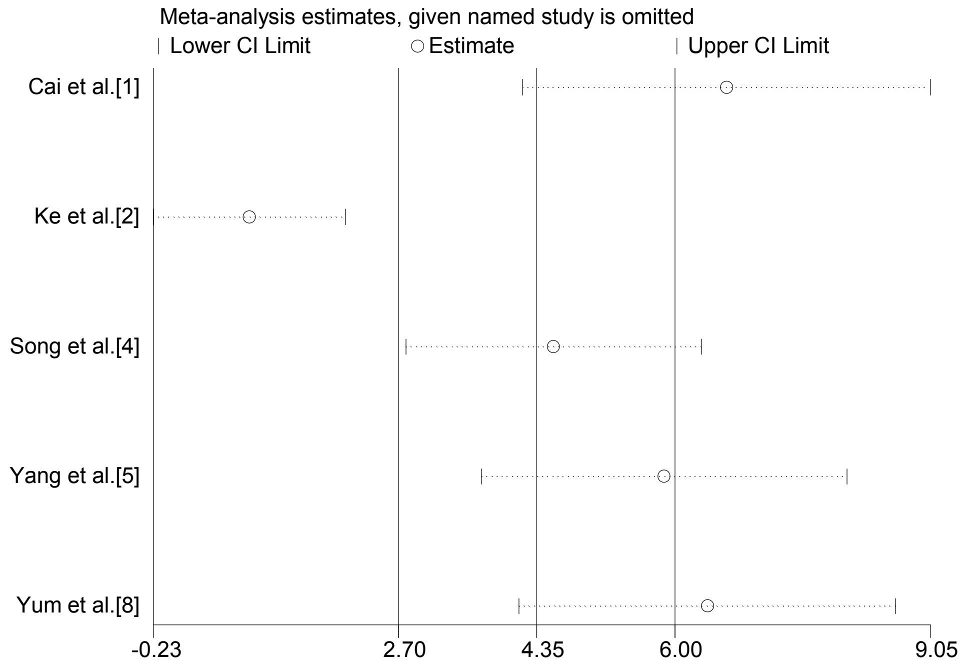

Figure S4. Serum DBP concentration.

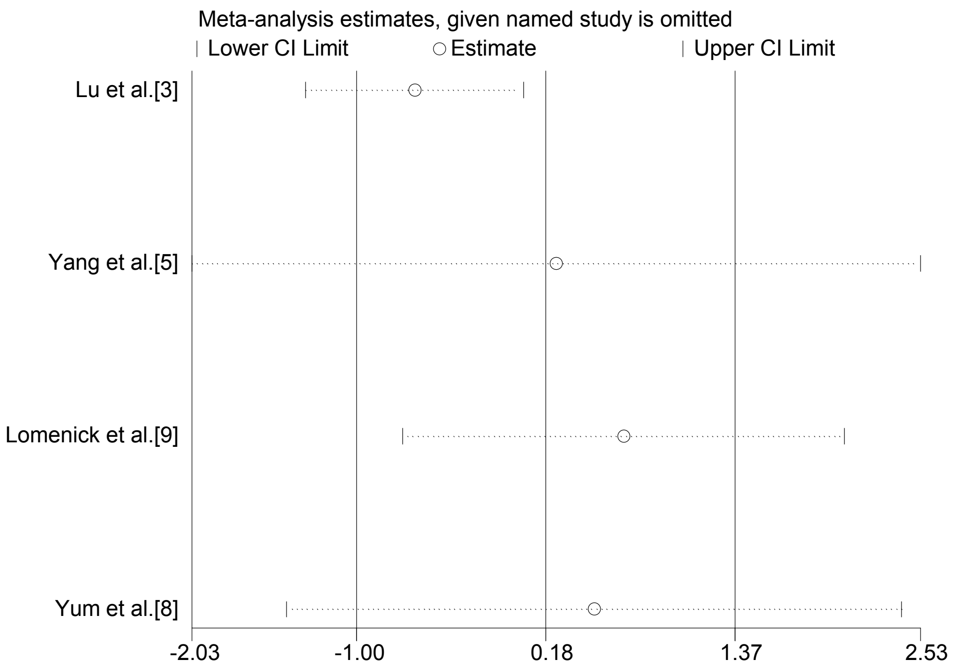

Figure S5. Serum MEHP concentration.

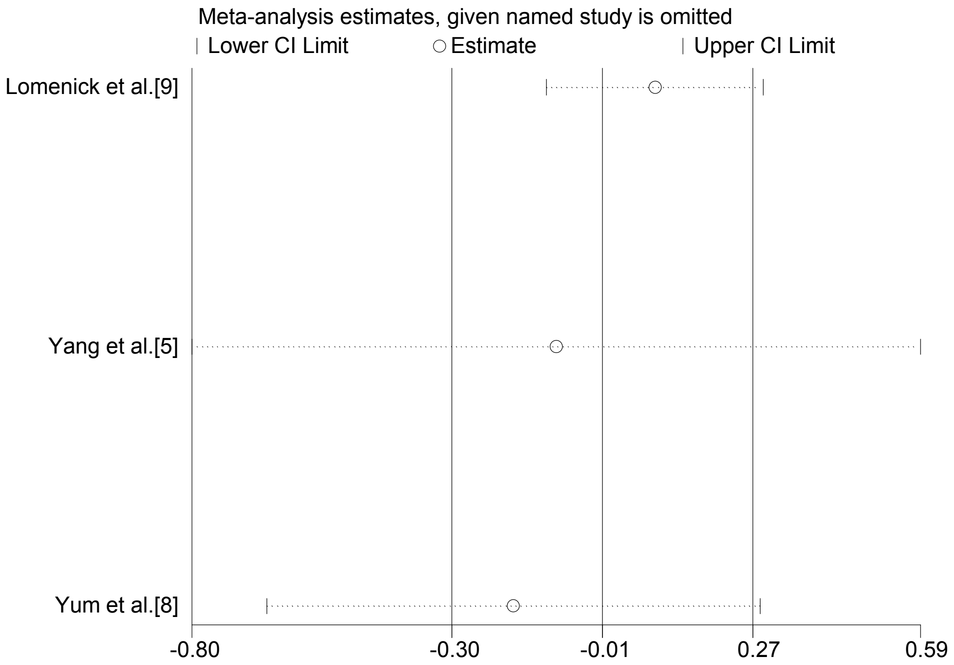

Figure S6. Serum MBP concentration.

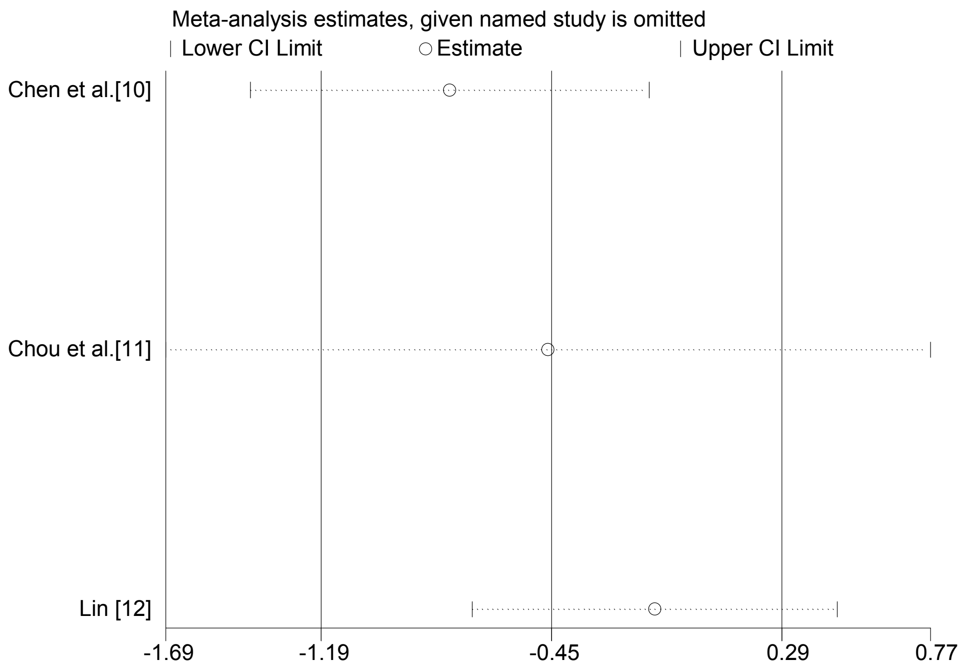

Figure S7. Urinary MEHP concentration.

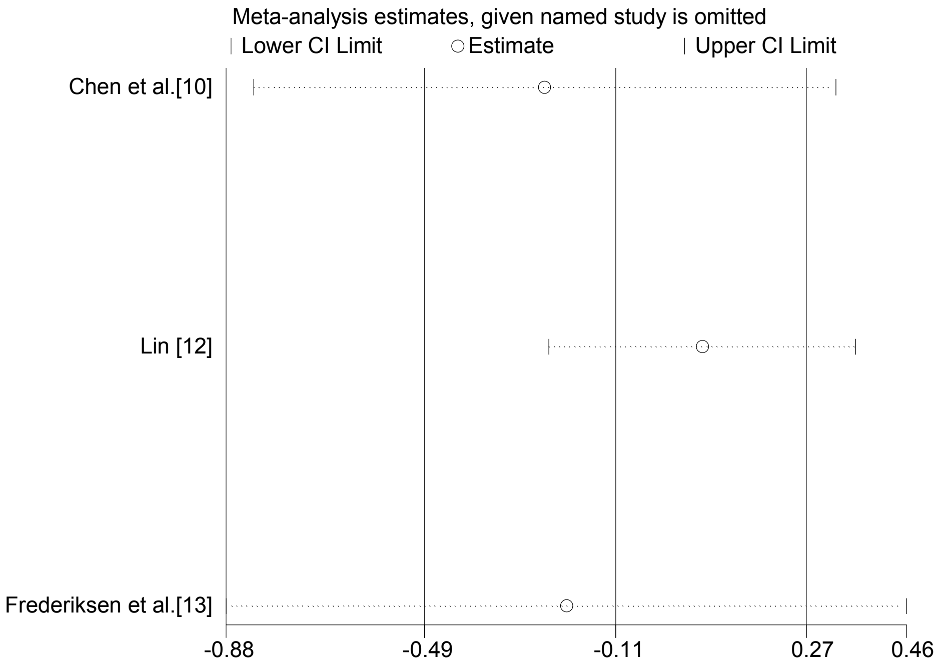

Figure S8. Urinary MBP concentration.

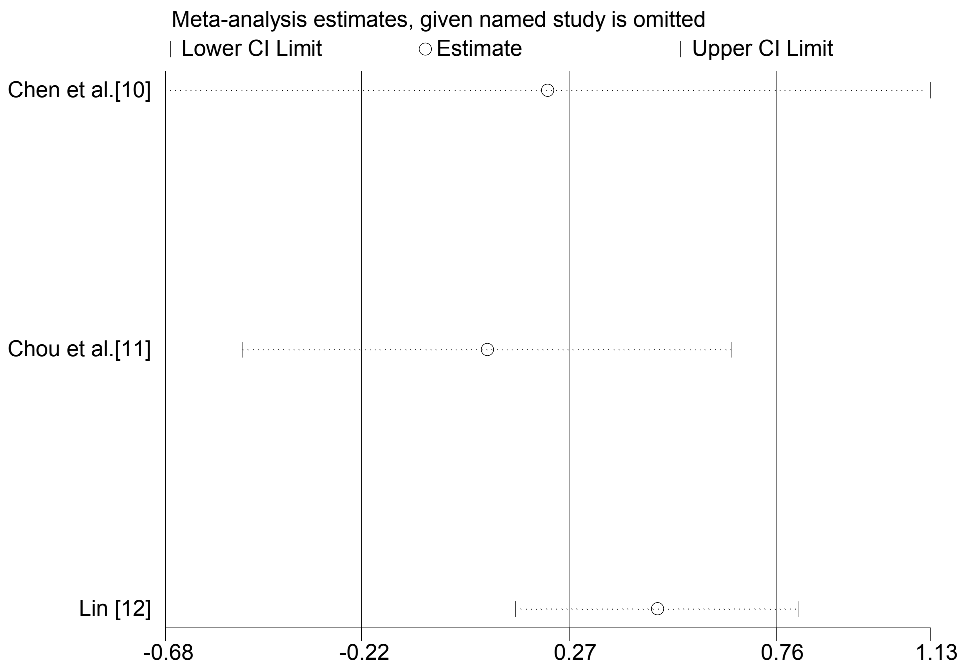

Figure S9. Urinary MMP concentration.

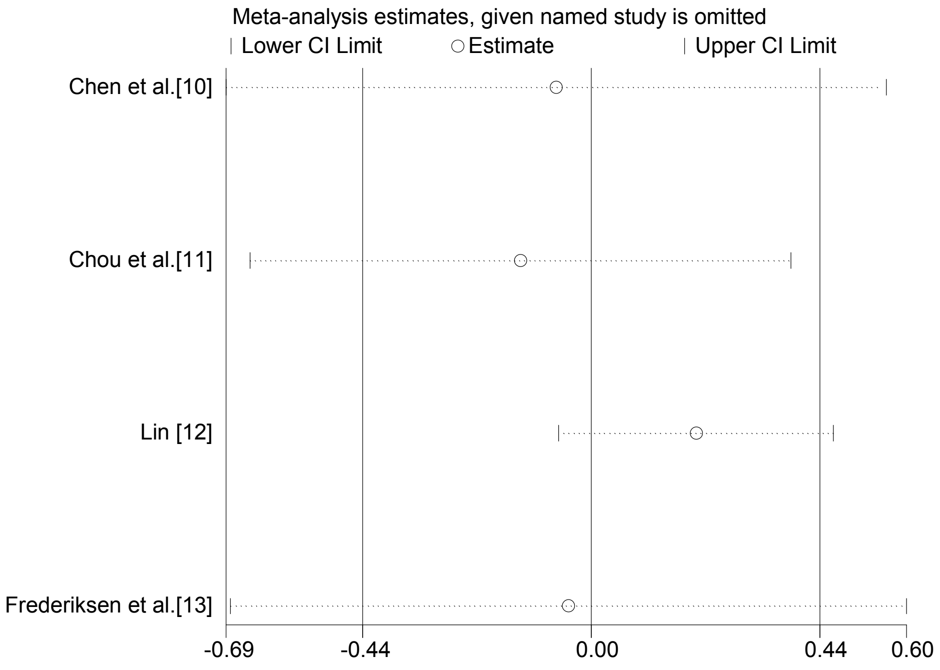

Figure S10. Urinary MBzP concentration.

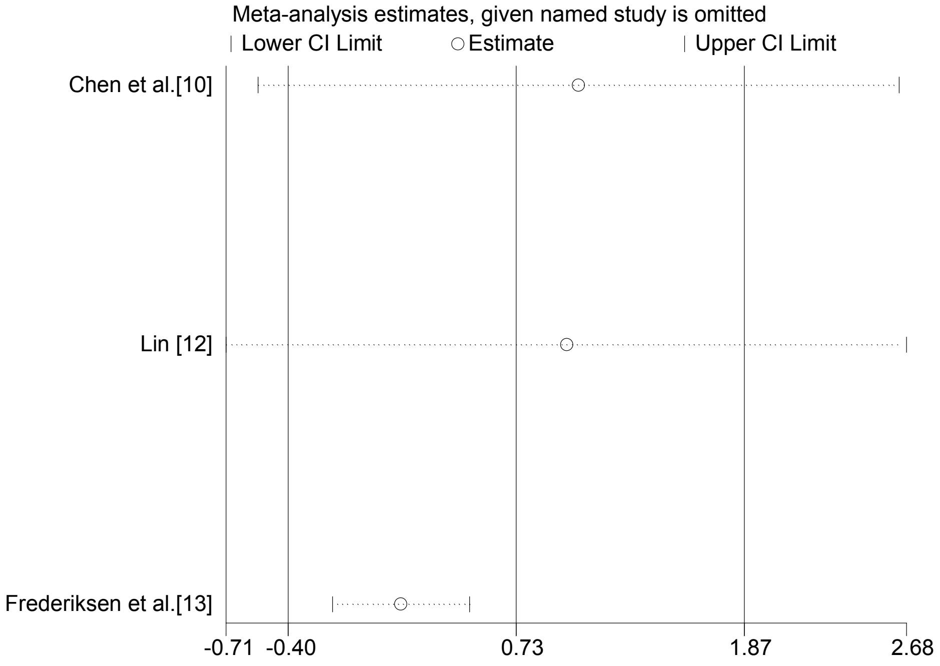

Figure S11. Urinary MEP concentration.

References

1. Cai, D.P.; Qiao, L.L.; Zheng, L. The relationship of the environment endocrine disruptors with precocious puberty. *Shanghai Med.* **2013**, *34*, 26–30.
2. Ke, J.; Yang, Y.; Duan, R. Study on effect relationship between estrogen receptor α gen polymorphism and environmental endocrine disruptors in precocious girls. *Matern. Child Health Care China* **2013**, *28*, 4184–4187.
3. Lu, J.P.; Zheng, L.X.; Cai, D.P. Determination of four kinds of environmental endocrine disruptors in blood serum of precocious puberty patients by high performance liquid chromatography. *J. Hyg. Res*. **2006**, *35*, 288–290.
4. Song, S.H.; Shan, L.L.; Ye, F.L.; Mei, Y. Application of gas chromatography in the detection of DEHP and DBP in human serum. *Med. J. Wuhan Univ. China* **2014**, *35*, 381–385.
5. Yang, Z.P.; Jian, Q.; Zhou, B.; Liu, S.Y.; Ding, G.Q.; Zhu, Y.M. Environmental Endocrine Disruptors in Serum of Sexually Precocious Girls. *J. Environ. Occup. Med.* **2014**, *31*, 331–335.
6. Zhang, S.L.; Wei, H.Y.; Gu, Q.R. Analysis on the correlation between environmental endocrine disrupter chemicals and childhood precocious puberty. *Matern. Child Health Care China* **2015**, *30*, 736–738.
7. Colón, I.; Caro, D.; Bourdony, C.J.; Rosario, O. Identification of phthalate esters in the serum of young Puerto Rican girls with prematurebreast development. *Environ Health Perspect*. **2000**, *108*, 895–900.
8. Yum, T.; Lee, S.; Kim, Y. Association between precocious puberty and some endocrine disruptors in human plasma. *J. Environ. Sci. Health A Tox Hazard Subst. Environ. Eng.* **2013**, *48*, 912–917.

9. Lomenick, J.P.; Calafat, A.M.; Melguizo Castro, M.S.; Mier, R.; Stenger, P.; Foster, M.B.; Wintergerst, K.A. Phthalate exposure and precocious puberty in females. *J. Pediatr*. **2010**, *156*, 221–225.
10. Chen, C.Y.; Chou, Y.Y.; Wu, Y.M.; Lin, C.C.; Lin, S.J.; Lee, C.C. Phthalates may promote female puberty by increasing kisspeptin activity. *Hum. Reprod.* **2013**, *28*, 2765–2773.
11. Chou, Y.Y.; Huang, P.C.; Lee, C.C.; Wu, M.H.; Lin, S.J. Phthalate exposure in girls during early puberty. *J. Pediatr. Endocrinol. Metab.* **2009**, *22*, 69–77.
12. Lin, Q. The Study on Relationship Urinary Phthalate Monoesters and house Dust Exposure for Precocious Puberty; National Cheng Kung University: Tainan City, China, 2010.
13. Frederiksen, H.; Sørensen, K.; Mouritsen, A.; Aksglaede, L.; Hagen, C.P.; Petersen, J.H.; Skakkebaek, N.E.; Andersson, A.M.; Juul, A. High urinary phthalate concentration associated with delayed pubarche in girls. *Int. J. Androl*. **2012**, *35*, 216–226.

© 2015 by the authors; licensee MDPI, Basel, Switzerland. This article is an open access article distributed under the terms and conditions of the Creative Commons Attribution license (http://creativecommons.org/licenses/by/4.0/).
